# Rice farmers’ knowledge, attitudes and practices towards mosquitoes in irrigation schemes in Côte d’Ivoire: a qualitative study

**DOI:** 10.1186/s12936-023-04785-y

**Published:** 2023-11-16

**Authors:** Kallista Chan, Kouadio Aimé-Charles Konan, Dimi Théodore Doudou, Ghislain Brou Kouadio, Jo Lines, Robert Aunger, Raphael N’Guessan, Lucy S. Tusting

**Affiliations:** 1 Department of Disease Control, London School of Hygiene & Tropical Medicine, Keppel Street, London, UK; 2 Centre de Recherche Pour le Développement (CRD)/Laboratoire de Santé, Société et Développement, Université Alassane Ouattara, BP 01 V 18, Bouaké, Côte d’Ivoire; 3 Institut Pierre Richet, Bouaké, Côte d’Ivoire

**Keywords:** Malaria, Community perceptions, Rice farming, Irrigation agriculture, Côte d’Ivoire, Sub-Saharan Africa

## Abstract

**Background:**

Irrigated rice cultivation in sub-Saharan Africa not only brings more malaria vectors to nearby communities, but also greater malaria risk. To aid the implementation of mosquito control in rice-growing communities, it is necessary to understand how farmers understand, view and manage their responsibility in mosquito generation and whether they are interested in coordinating to minimize it.

**Methods:**

Qualitative methods (observation grids, semi-structured in-depth interviews and focus group discussions) were used to reveal the perceptions of mosquitoes and their control in two irrigated rice farming communities in central Côte d’Ivoire near the M’bé and Lokapli irrigation schemes.

**Results:**

All rice farmers viewed mosquitoes as severe nuisances, and most acknowledged that they caused *djèkouadjo* (malaria) and were less numerous during harmattan (dry season). Many study participants believed that mosquitoes originated from grasses and stagnant water around villages. Only those living closer in proximity (~ 1 km) to the paddies believed that mosquitoes came from the *bas-fonds* (irrigated lowlands). However, they did not associate mosquito production with rice cultivation. Some farmers believed that there were more mosquitoes in recent years than historically because of the dam construction, but remarked on the importance of the dam (and bas-fonds) for their livelihood. Many farmers were not convinced that mosquito control could occur at farm-level.

**Conclusions:**

To enhance accountability amongst rice farmers, there is a need for greater awareness on the rice-mosquito link, and emphasis that the link does not imply a trade-off between food production and health. Training should not only be directed towards farming communities, but also agricultural and health extension workers. Future riceland mosquito control methods must focus on improving crop productivity and address collective action problems that may occur.

**Supplementary Information:**

The online version contains supplementary material available at 10.1186/s12936-023-04785-y.

## Background

Malaria remains a major health problem worldwide, with an estimated 247 million cases in 2021 [1]. Although nearly half of the world’s population is at risk of malaria, sub-Saharan Africa (SSA) carries a disproportionately high share of over 95% morbidity and mortality. With around 7.4 million cases in 2021, Côte d’Ivoire is the 10th highest burdened country [1].

Malaria has complex associations with agriculture [2, 3]. In SSA, it has major links with irrigated rice [2, 3]. Rice paddies, due to their flooded nature, provide excellent and stable breeding sites for mosquitoes to thrive and proliferate [4]. Accordingly, compared to neighbouring non-rice-growing areas, communities located near irrigated rice cultivation are exposed to sixfold higher adult malaria vector abundances and twofold higher malaria transmission [3]. In southwest Nigeria, it was estimated that rice farmers lose 10 days per year due to malaria, where a small proportion of farmers even indicated more than 20 days lost to malaria [5]. As a result, malaria influences agriculture too. Through the disruption of rice operations (labour loss), the inability to engage in intensive farming practices, and the high expenditures on malaria treatment, farmers achieve lower yield returns and less agricultural investments [6, 7]. Thus, despite the advantages of developing water resources for agricultural purposes, these investments can have adverse effects on the health and physical, social, and economic wellbeing of households and, sequentially, their agricultural productivity [8]. This reinforces the need to control mosquitoes in agricultural communities so that farmer livelihood (and the overall development of the economy) is not hampered by malaria.

However, rice cultivation, especially irrigated rice, remains an important strategy across SSA to improve food security and keep up with ever-increasing consumer demands. Currently, there are goals in place for African countries to double rice production to 56 million tonnes by 2030 [9]. In Côte d’Ivoire, one of the priorities in the national rice development strategy involves the expansion of irrigated rice cultivation [10]. These strategies overlook the associations between rice and malaria. Whilst agricultural development agencies are actively promoting major rice expansion, health development agencies are planning for malaria elimination. This clash of equally important development goals necessitates methods of rice cultivation that can minimize mosquito proliferation. Methods of adult vector control such as the use of long-lasting insecticidal nets and indoor residual spraying near rice communities should be maintained, but they are neither permanent nor complete solutions [11]. There is instead a need for supplementary vector control methods such as larval source management, particularly through environmental management, to prevent vector production in the first instance.

Smallholder farmers constitute most of the rice production in SSA [12]. Thus, for any riceland mosquito control strategy to succeed, cooperation from all rice farmers in an irrigation scheme would be required. If a portion of rice farmers failed to adopt an intervention, mosquito production, although reduced, would not be eliminated. This is a case of the “n-person prisoner’s dilemma”, where collective participation of a new practice is a prerequisite for achieving a goal from which all individuals benefit [13]. Sometimes, individuals do not cooperate due to conflicting interests or in order to enjoy a “free ride”. This is related to a prominent and pervasive public health problem, the “collective action problem” [14]. Consequently, it is essential to involve rice farming communities in the process of designing and implementing potential control methods.

Heightened awareness on the link between rice and mosquitoes amongst farmers is also necessary. Rice farmers that are aware of this link seem to be more willing to adopt and practice farm-level mosquito control. In Rwanda, 92% of farmers recognized that rice cultivation contributed to malaria and were hence willing to spend 1–2 h a week on larvicide (*Bti*) application [15]. Ingabire et al*.* [16] also established in Rwanda that farmers that were knowledgeable about malaria, were involved in rice cultivation for less than 15 years and perceived rice farming as less profitable were more likely to contribute time to *Bti* applications.

Numerous studies have explored rice farmers’ knowledge, views and perspectives on malaria, its aetiology, its symptoms, and (adult) vector control practices [5, 6, 8, 17–23]. However, these investigations were often limited to the simple acknowledgement that malaria was transmitted by mosquitoes. Except for a few studies, rice farmers’ views and opinions on mosquitoes, their origin, and their links with rice were rarely investigated [16, 24–28].

To aid the implementation of malaria vector control methods in rice communities, it is necessary to understand whether farmers are aware that their fields generate mosquitoes, whether they are concerned about it or feel any responsibility towards it and whether they are interested in coordinating to solve the problem. Thus, this study seeks to examine local rice farmers’ knowledge, attitudes/perceptions and practices/behaviour about mosquitoes and to determine if there are any existing or potential collective initiatives for riceland malaria vector control in two rural rice communities in central Côte d’Ivoire.

## Methods

### Study area

**Fig. 1:**
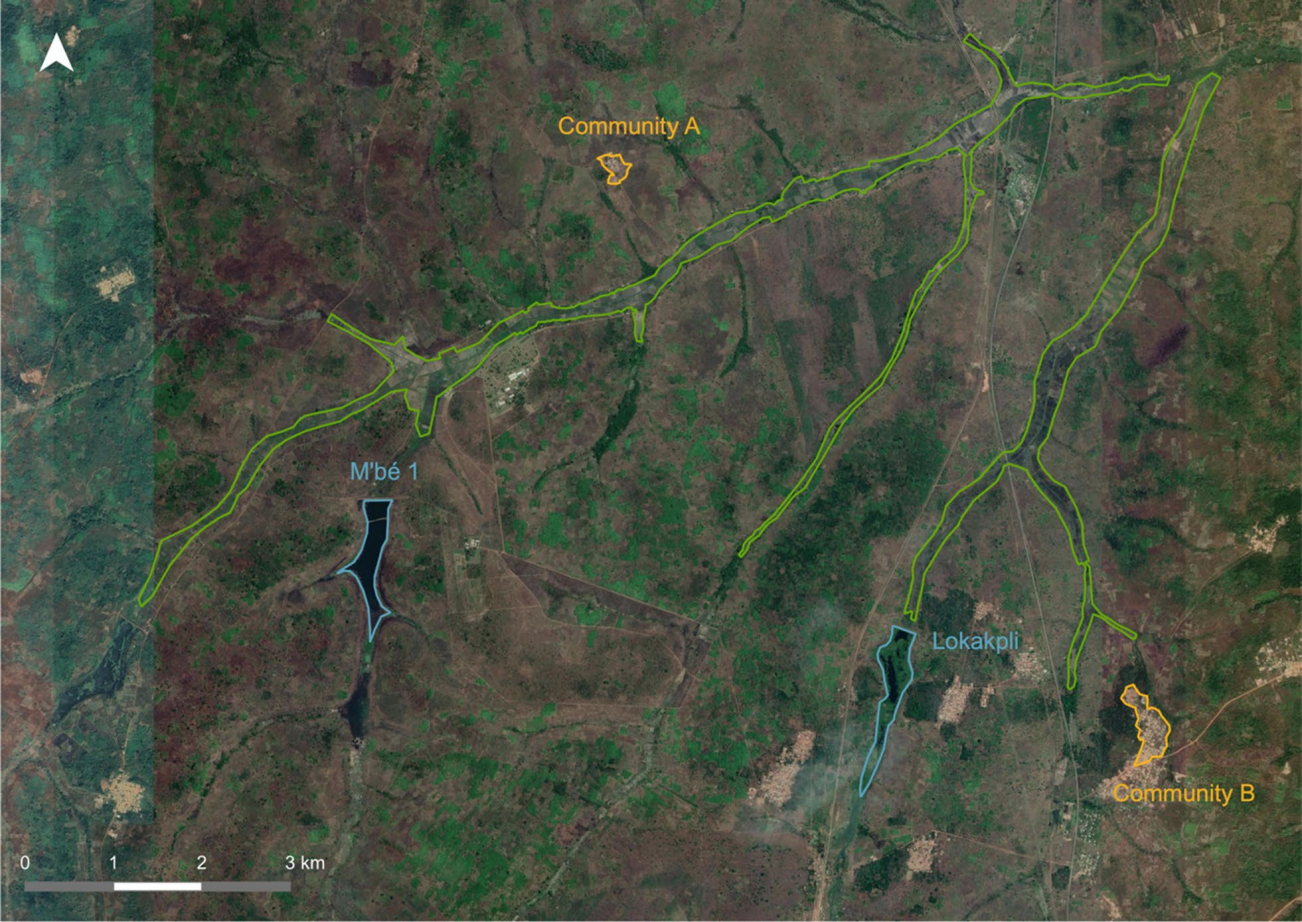
The two study sites and their corresponding dams and irrigation schemes

This study was conducted in 2021 in two rural communities, Community A and Community B, 20–30 kms north of Bouaké (and around 9 kms apart), which is situated in the central region of Côte d’Ivoire. Community A is a small village, deprived of electricity, of around 200 people [29]. The main economic activity of Community A is irrigated rice farming with two cropping cycles, where they use the M’bé-1 dam and irrigation scheme. The village is situated around 1 km away from rice fields (Fig. 1). Community B is a larger village of around 700 people and is part of a peri-urban town [29]. Its main economic activities are yam and rice cultivation. Community B is situated less than 2 km away from neighbouring rice fields, but farmers’ fields were usually located in another part of the Lokapli irrigation scheme, around 4 km away from the village. These two communities were purposely chosen because of their proximity to their rice growing areas, their local language (Baoulé), and their sociodemographic differences.

The two study villages are located in the equatorial transition climatic zone, where seasons are distinguished by a long rainy and long dry season [30]. The rainy season occurs from April to October, where rainfall reaches its maximum in June (30–40 mm) and September (45–55 mm). The dry season occurs from November to March, marked by *harmattan,* which is characterized by hot and dry trade winds blowing from the Sahara over West Africa. According to routine health service statistics, this study region had a malaria incidence of 166 cases per 1000 children under 5 in 2019 [31].

### Data collection methods

This study used a three-stage approach to cover different knowledge, perceptions and behaviours in each community: observation method, semi-structured in-depth interviews (IDIs) and focus group discussions (FGDs). All three approaches were conducted by KACK (a PhD candidate in social science and native speaker in Baoulé) alongside a supervising moderator from September to December 2021, across both rainy and dry seasons. Collection instruments for all three approaches (Additional files 1 and 2) were provided to KACK. Preliminary tests of the IDIs were conducted in early April and amended accordingly in May. IDIs and FGDs, both conducted in the local language (Baoulé), were audio recorded with permission from interviewees.

#### Observation grid

Ethnographic immersions were conducted for 1 month at each village. Observation grids, which are guides to remind the observer the topics of interest, were used to record information on rice cultivation and mosquitoes within domestic spaces and rice farms. The following aspects were recorded: behaviour towards mosquitoes, behaviours favouring or reducing mosquito proliferation (including mosquito control practices), sleeping habits, population movement, cultural practices with rice and cases of free-rider problems.

#### Semi-structured in-depth interviews

Qualitative semi-structured in-depth interviews (IDI) were administered to up to 25 rice farmers and/or their family members in each community. They were used to assess the beliefs, opinions, views, perspectives and behaviours of rice farmers on (1) the advantages and disadvantages of rice cultivation, and (2) the following aspects about mosquitoes: (a) their origin, (b) their occurrence, (c) the severity of the problem in terms of nuisance and/or disease transmission, and (d) behaviours or practices perceived to favour or reduce their proliferation. The atmosphere and non-verbal behaviour made during each interview were also recorded. An interview guide is presented in Additional file 1.

Participants were selected based on the level of compliance as well as observations noted during the ethnographic immersions. No distinctions were made with respect to gender nor age; community members above 18 years old from a rice-cultivating homestead could be enrolled for interviews.

#### Focus group discussions

Five (more or less) homogenous groups of ten rice farmers, separated into women, men, and youth groups (individuals under 30 years of age), from two villages were assembled in open domestic spaces for focus group discussions (FGDs). Individuals were classified by gender and age in order to create a comfortable environment where all participants can feel free to speak openly about their shared experiences. A total of 10 participants per group was selected as the appropriate sample size because a larger group may limit the detail of responses whilst a smaller group may cause uncomfortable pressure to talk. First, information on mosquitoes, as revealed by the IDIs, and views and perspectives of the general population’s responsibility in mosquito production were discussed. Second, if a link between rice cultivation and mosquito production was correctly established, discussions on the existing collective practices were conducted as a community to solve this issue, the strengths, and weaknesses of said practices and reasons for their success or failure. Participatory action research (PAR) tools were used to aid focus groups in their exploration to improve existing collective practices against riceland mosquitoes. Alternatively, if a link between rice cultivation and mosquito production had not been established, PAR tools were used to raise awareness about the link and aid the groups in identifying actions that must be carried out collectively to solve the problem. The main PAR tool used was mapping, where the focus group describes the (physical features of the) territory they use and the resources they use for livelihood activities [32]. A supervising moderator was present to help direct discussions in case some topics were not well covered; probes for the FGDs are presented in Additional file 2.

### Data analysis

Audio recordings from IDIs and FGDs were transcribed in Baoulé and translated to French by local university graduates. The transcriptions were sequentially translated to English by KC, and thematically analysed using NVivo (version 12). A coding framework was developed based on themes which emerged from the data, where the data from each participant were coded by KC and JL and discussed with the other co-authors. Key themes and their examples were then presented in vignettes and direct quotes.

## Results

A total of 43 participants were recruited in the IDIs: 25 in Community A and 18 in Community B. A total of 50 participants were recruited in the FGDs: three groups of 10 youth, female and male participants in Community A and two groups of 10 female and male participants in Community B. There were no refusals to participate. Quotes from participants are cited with fictitious initials to maintain anonymity.

### Rice farming: characteristics and experiences

In both communities, all rice farmers cultivated rice in irrigated lowlands, where 6 of 43 (14%) had their plots close to dams. Four farmers also cultivated rainfed rice near the river. Two-thirds of the participants cultivated other crops alongside rice, such as yams, cashews, maize, and market gardening (cucumbers, tomatoes, and okra).

Rice plots were an average size of 1.5 hectares in Community A and 0.9 hectares in Community B. Farmers had been cultivating rice for an average of 11.8 years, ranging between a few months to 38 years. Almost everyone grew the WITA-9 variety, but some also planted GT-11 and C-26. Farmers had previously tried other varieties (e.g. Bouake-189, Orilux-6) but switched because current varieties were more resilient to insects, diseases, and the dry season (harmattan) and so produced greater yield (higher profitability). Some farmers mentioned that the variety they chose to grow also depended on seed availability and market demand. Two farmers from Community A also stated that researchers from the neighbouring rice research institute AfricaRice “*advised [them] to stop using older varieties and recommended WITA-9*” instead.

**Fig. 2:**
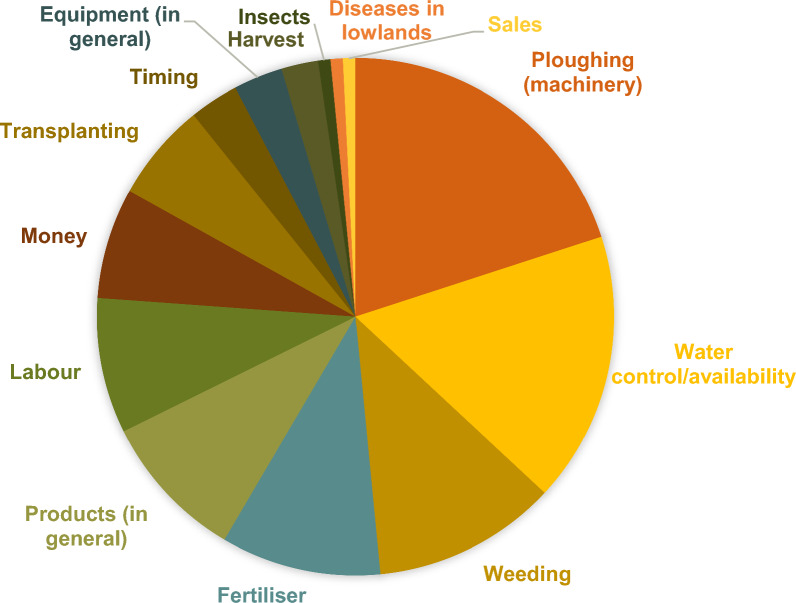
The perceived difficulties in rice farming

When asked about the ease and difficulties of rice farming, only one participant stated that there were no difficulties: “*rice cultivation is work that nourishes the child—it brings money and allows [him] to send children to school*”. Some respondents reasoned that the ease of rice cultivation depended on whether one had enough means, i.e., money to purchase products and hire labour and machinery for ploughing. Most farmers (n = 32) said that rice farming was difficult because of its many requirements: machinery for ploughing, water control, inputs (herbicides, fertilisers, pesticides), and labour for transplanting and weeding (Fig. 2).

Most farmers indicated ploughing as the main issue, as machinery availability was limited; the walking tractor often broke down and belonged to other villages of another ethnic group which prioritized their own communities. The second most frequently cited issue was water availability. The dam used by farmers in Community A was operated by AfricaRice, and so farmers lacked control and there was sometimes resistance in opening water channels during the dry season. In Community B, respondents complained about water scarcity during the dry season due to poorly maintained canals; overgrown with grasses, that blocked water flow to rice fields farther away from the dam. Some disclosed that this issue created arguments between farmers whilst others pointed out that the president of the rice cooperative should organize these regular collective cleaning sessions. Many farmers pointed out the imbalance in effort towards cleaning:

*“The water problem is like witchcraft. Even at midnight, I am still there…I block the water [flowing to other plots] so that the water goes to my fields”.—MJLK*

*“There are some people who are difficult, who open their pipes and never close them. There are others who don’t maintain their canals, so they are full of weeds. Every year we will clean up but when you mention it to them, they don’t listen to you”.—NKJP*

*“The president of the cooperative has to give orders for a time to clean the canals. We inform everyone but some do not go. You who are [far away from the dams] clean up properly for yourself but those who are close to the dam don’t do it, so you must leave your fields and go to theirs to clean it for them…we don’t love each other—it is wickedness!”—SNR*

In terms of the perceived disadvantages of living near rice fields, there were some differences between communities. Whilst most respondents from Community A cited mosquitoes (14/25, 56%), sometimes together with the cold (6/25, 24%), many (10/25, 40%) also thought that despite them, it was an advantage to be closer to their workplace, saving on transport expenses. This was pointed out by one participant:

“Living next to the bas-fonds and the rice fields, our village has never lacked mosquitoes. We are always under attack. Even all the villages nearby call us the mosquito village, but luckily, we have easy access to the rice fields”.*—*MRCL.

When some respondents were prompted about health, the majority did not think that living near rice fields led to more illnesses. Only a few did, specifying Guinea worm disease (note that, as of 2013, Côte d’Ivoire was certified free of Guinea worm disease [33]), mosquito-borne diseases, and cancer. Of 25 participants from Community A, only four (16%) stated that it was not bothersome living next to rice fields.

In Community B, more than half of the farmers (10/18, 55.6%) declared that living near rice fields was not troublesome, many of whom (6/10, 60%) said it would be more convenient. A third of the farmers still cited mosquitoes as a problem and a few mentioned that living near the *bas-fonds* could bring illnesses such as Buruli ulcer, malaria, and African trypanosomiasis. Two farmers did not perceive it as a danger to health, where one said:

*“If it made us sick, we who have been in the* bas-fonds *since a long time would all have died”.—MORY*

### Mosquitoes: knowledge, attitude, and perceptions

#### Problems caused by mosquitoes

When asked if their village had any mosquitoes, all participants replied “*yes*”, where a third of them added comments to the effect of “*in abundance/numerous/too much!*” and a few exclaiming or laughing in disbelief at the question. Many farmers expressed without further prompt that mosquitoes were a significant problem mainly because they cause nuisance. They specified that mosquitoes disturbed sleep (which caused fatigue, weakness, and illnesses) through noise and/or bites, forced villagers to wear long sleeved clothing, jackets, and boots as personal protection, and prevented evening activities such as going outside, trade, and studying for children. Three respondents also mentioned that mosquitoes necessitated bed net use, which in turn were uncomfortable or inconvenient to use. Most farmers (33/43, 76.7%) stated that mosquitoes can lead to diseases such as *djèkouadjo* (the local name for malaria, n = 26 [60.5%]), zoonotic diseases (n = 4), diabetes (n = 1), AIDS (n = 1) and anaemia (n = 1), which led to treatment costs, hospitalization, and death. When asked to rate mosquitoes against other common insects such as flies and bedbugs, mosquitoes were consistently ranked the worst by all respondents. The following quotations demonstrate what a few participants think of mosquitoes:

*“If there were no mosquito nets, I would leave the village. That's the only solution.”—NBL*

*“Yes, [mosquitoes are annoying] because we are not free! We do not live peacefully. My body can’t stand the heat, but if I don’t use mosquito nets, the mosquitoes will start biting me and I’ll get malaria”.—MRCL*

*“Mosquitoes are worse than working in rice”.—KOH*

During the ethnographic immersion, it was recorded that villagers often complained about mosquitoes in the evening, tended to go to bed early and ordered their children to sleep early (under the bed net) to avoid catching malaria. In the field, farmers were observed to rush home in the evening to evade the mosquitoes. Some farmers claimed that the mosquitoes in the field were larger than those in the village.

#### Perceived origin of mosquitoes

Almost everyone across the two communities speculated that mosquitoes originated from neighbouring grasses or bushes, followed by wastewater (Table 1). Following these two sites, the most common speculations differed by communities. In Community A, farmers attributed mosquitoes to *bas-fonds* (see Additional file 3), stagnant water, and the edge of water bodies (*bas-fonds*, dams, and rivers). Farmers referred to the *bas-fonds* mainly when mentioning their experience working in the fields at night:

*“Even at night, you can’t go to the* bas-fonds*, everywhere is full of mosquitoes”.—CGK*

*“When you go to the field at night, the way the mosquitoes bite you are different from when you are in the village. In the village, they don’t bite you like that”.—NKJP*

*“Rice is a type of grass too—all the mosquitoes are in [the plants]. When you are there, there are mosquitoes hitting you, it’s like you’re getting stoned!”—SNR*

**Table 1:** The perceived origin of mosquitoes, enumerated by number of mentions in IDIs

Ranking	Community A	Community B
1	Grasses/bushes (20)	Grasses/bushes (15)
2	Wastewater (11)	Wastewater (10)
3	*Bas-fonds* (10)	Stagnant water (9)
4	Stagnant water (8)	Garbage (6) and river (6)
5	Edge of water bodies (6)	Dam (4)
6	Garbage (4)	Mangoes (3)
7	Forest (3) and dam (3), dark (3), humid (3)	Forest (2) and God (2)
8	River (2), animals (2)	*Bas-fonds* (1)
9	Agricultural ponds (1), ploughing (1), and God (1)	

In Community B, mosquitoes were more often attributed to stagnant water, garbage, and rivers; *bas-fonds* were only mentioned once. Similar patterns in both communities could be seen from mapping conducted in FGDs: the *bas-fonds*, dams and wastewater were perceived to be main mosquito breeding sites in Community A whereas rice fields were ranked lower in Community B (Table 2). Several participants across both communities acknowledged that mosquitoes can migrate, namely from the *bas-fonds*, dams, and rivers as well as grasses, wet and dirty places. Conversely, a few said that mosquitoes could not migrate from dams because they were too far away or that mosquitoes only came from within the village.

**Table 2:**
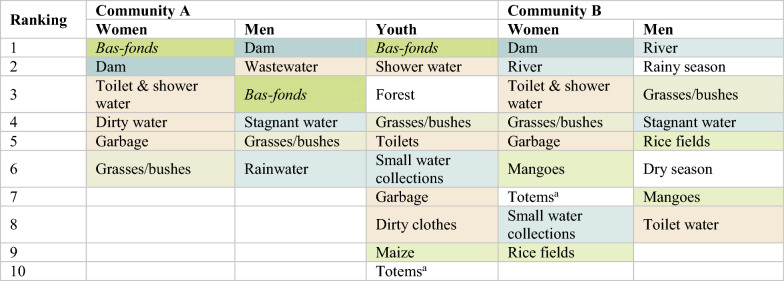
The perceived origin of mosquitoes as ranked in the five FGDs (number of participants = 50)

^a^Totems refer to divine retribution from God for the disobedient who have trivialised ancestral practices and prohibitions

Most participants were aware that mosquitoes developed in water. Rare exemptions included beliefs that mosquito development occurred in grasses and/or was only favoured by water or humidity. However, none of the respondents stated that there is a link between mosquitoes and rice cultivation. When probed, the majority (30/43, 69.8%) of the participants did not believe it existed. About half (14/30, 46.7%) of this majority specified that mosquitoes were linked to *bas-fonds* but not to rice itself (since rice fields are part of the *bas-fonds*). One farmer believed that mosquitoes could not survive in rice fields because agricultural insecticides are regularly sprayed. Many farmers also explained that mosquitoes were present even when rice was not being cultivated in the *bas-fonds*. Similar comments were gathered across all five FGDs. In IDIs, 11 farmers agreed that mosquitoes were associated with rice, where one mentioned that mosquitoes were associated with ploughing, another with transplantation and some thought that since it resembled grasses, rice could attract mosquitoes. The following statements capture the beliefs of a few farmers on the link between rice and mosquitoes:

*“I say it is the water at the bottom of the rice that attracts mosquitoes, but the rice [plant] will not attract mosquitoes”.—GNM*

*“Rice can’t bring mosquitoes. The mosquitoes were already around before the forest was cleared and rice was grown. They have always been around, but not as numerous. We know that rice does not cause mosquitoes. There are times when there is no rice in the bas-fonds, but the mosquitoes are still there. So, rice can't be a problem.”—SYP*

*“If you stir the rice, you will see mosquitoes flying away. Going to the fields at night—it is not worth it. If you want a tonne [of mosquitoes], you can find it”.—MORY*

*“We can’t forbid rice growing just because the mosquitoes are going to kill us. What are we going to eat? …Thanks to the fields, we can harvest some rice for children to get educated and a little for us to eat. It’s not easy (laughs)!”—MRCL*

#### Occurrence of mosquitoes

When asked about the timing of mosquitoes during a 24-h period, almost all respondents said that mosquitoes started arriving to the villages between 18.00 and 19.00 h in the evening and were present until dawn and morning.

When asked about the timing of mosquitoes annually, all farmers discerned that mosquitoes were not as numerous from December to February (harmattan) but were abundant from March to October (the rainy season). For the latter, respondents often associated mosquitoes with heat, grass, and the mango season. For the former, many participants speculated that burning bushes, the wind and/or the cold during harmattan kept mosquitoes away. Overall, however, none of the respondents said that there was a period within a year without mosquitoes:

*“A time without mosquitoes does not exist! Even during the harmattan period, they decrease but they are there.”—SYP*

When asked about the historical differences in mosquito abundance in the past few decades, 23 participants (53.5%) said that there were more mosquitoes nowadays (that “*when they were younger, they could sleep without mosquito nets”*), 11 (25.6%) said there were more in the past and five (11.6%) said that there were no differences. Of the 23 farmers that believed that there were more mosquitoes currently, nine (39.1%) attributed it to the construction of a nearby dam and often with deliberated its importance for their livelihood. Five (21.7%) attributed the increase of mosquitoes to the presence of more grass, garbage, and wastewater and three (13%) to the loss of traditions (that since Christianity, the community no longer followed ancient laws and, therefore, the community is now paying the price). The following quotations summarize their thoughts:

*“I myself cannot understand. Our parents told us that the mosquitoes weren't around too much before... Before, the neighbouring villages said that mosquitoes were abundant in Community A but nowadays there are mosquitoes in all these villages. Mosquitoes are everywhere now. Even in our [matriarchal] village, which is far from here, there are mosquitoes there. Except for houses that are in town where there is light and an air conditioning system. When the house is air-conditioned, they can't stand the cold and they leave to go elsewhere.”—REMI*

*“People before respected the [traditional] prohibitions, but now no one respects them.”—RLND*

*“Mosquitoes have been here a long time. We don't know what sent them. When they made the dam, the mosquitoes multiplied. Now they sting much compared to the past… Because the dam is close to us and we are on the edge of this big bas-fonds there and there are also small bas-fonds around the village. When the rainy season comes and the water stagnates everywhere, mosquitoes come in force. It's the stagnant water everywhere that matters, but without water we can't do anything. If we don't want the water to stagnate too, what are we going to eat? It is because of the bas-fonds that mosquitoes have the strength.”—Man from Community A FGD*

However, two farmers believed that recent mosquito proliferation was not because of the dam:

*“They made the dam in 1997-98 so, I can’t say it is because of the dam. Because the mosquitoes started to tire us long before we started the dam.”—NKJP*

Of the 11 respondents who said that there were more mosquitoes in the past, 4 rationalized it with the current distribution of mosquito nets, 3 with the fact that the nets are now impregnated with insecticide and 3 with how the village had grown in size so mosquitoes are spread across more community members, or their house was no longer at the edge of the village. Overall, a quarter of the participants also observed that there were no years without mosquitoes.

### Mosquito control practices

#### Household-level

At the household level, everyone who participated in the IDIs claimed bed net use. Many observed that nets were the most effective control against mosquitoes; insecticidal sprays (*Timor* and *Rambo*) were popular (n = 24, 55.8%) but farmers claimed that they could only “*calm mosquitoes down a bit”* and bought them only if they had extra money. The effectiveness of these sprays is expressed here:

*“Timor is also good because it drives away mosquitoes automatically... It is the mosquito net that is more effective, because even if you pump the Timor they will come back. But with the mosquito net, they do not have access for entry”.—LKG*

*“Yes, [Timor] calms [the mosquitoes] down a bit, they go and then afterwards they come back again. But before the product we use to treat cotton there when you spray it, it kills certain insects. But now it just drives them away—it doesn’t kill them anymore”.—KKP*

Twelve participants (from 43 IDIs, 27.9%) also used coils (*Moskito*), seven regularly cleaned their house and yard and five ensured that their house was well-constructed without many window, door, and roof openings. One farmer in Community A suggested that having electricity for light and air conditioning would help control mosquitoes. There were slight differences in household mosquito control between the two communities. In Community A, most farmers used both nets and insecticidal sprays (despite scepticism in the spray’s efficacy), and sometimes coils. In Community B, most participants only used a net and cleaned their home.

From the ethnographic immersion, the field observer noticed that during the evening, villagers regularly swatted their legs with their hands or a piece of cloth, put their feet in bags, shared mosquito coils amongst each other or used a torch to kill mosquitoes. House screening was also installed in some homes, using tarpaulins or traditional cloth.

#### Village-level

At the village level, the majority of the IDIs (n = 30, 69.8%) and FGDs asserted that village cleanliness was required to control mosquitoes. Specifically, it entailed burning grass, removing garbage, and removing stagnant water and wastewater (from toilets, showers, and cooking) by making pits and closing their opening using slabs or by building proper showers. A few participants indicated that bush clean-ups should be organized by the youth president or that they would require machinery for ease. Compared to Community A, these “sanitation programmes” had been less successful in Community B due to disputes over land distribution and ownership. Most farmers therefore resorted to conducting clean-ups individually and more frequently, using machetes or herbicides.

Following village clean-ups, the most common answer was that there were no solutions against mosquitoes (n = 11, 25.6%); a few farmers claimed that cleaning was not effective. Five (11.6%) farmers suggested spraying the village with insecticides (i.e. insecticidal product distribution and aerial spraying by the government) but clarified that it was only a temporary solution and labour intensive. In an FGD between female rice farmers in Community A, it was established nothing had been done to reduce mosquitoes because the village “*did not have the money to hire a manager from town to reduce mosquitoes”.* The following quotations encapsulate many participants’ suggestions:

*“The village has to be clean, when the village is clean and they don't know where to land, where to breed, there won't be many of them there”.—KASS*

*“What we can do is respect each other—so that we can all work together to make the village clean… But there are many stubborn people that do not respect the laws of the village”.—IVN*

*“I don't see what we can do to reduce mosquitoes… Maybe it's products [the state is] going to send us, otherwise I can't see.”—TCHD*

*“Even if we spray insecticides, we can't spray the whole village.”—PIT*

Several participants from IDIs and FGDs also explained that the community had not actioned on reducing mosquitoes because they were paying penance for breaking traditional laws.

#### Farm-level

At the rice field level, a third of the IDI respondents did not observe links between mosquitoes and the *bas-fonds* or rice fields and hence did not reach this topic of conversation. Regardless, most farmers (17/43 IDIs, 39.5%) were not convinced that anything could be done to reduce mosquitoes in the *bas-fonds,* sometimes referring to personal protection (through long-sleeved clothing) or village-level control:

*“Over [in the field], we can’t do anything because the terrain is vast. There I do not see a solution because … we [farmers] are not in contact every day. But we in the village*

*“At the level of the bas-fonds, we do not have any solutions yet because if the bas-fonds are not there, it is difficult to eat. Currently rice feeds people—yams are no longer successful, and it is thanks to the bas-fonds that we can eat. Maybe it's the dam, but if we stop the dam, we can't eat”.—Young person from Community A FGD*

Seven farmers (16.3%) proposed insecticide application but similar to household-level observations, many were doubtful of how effective and manageable (daily application of) chemical control would be. One statement that encapsulates this was by MORY:

*“No, [we can’t do anything]! there is no solution for this, I can't buy Rambo to spray the rice… I also can't take the mosquito net to cover the rice!”.*

A few farmers, in both IDIs and FGDs, said that if they were shown or given the appropriate insecticides by the state, that they would do it:

*“No [we cannot reduce mosquitoes in rice fields], except if the state shows us drugs to reduce them”.—KRST*

*“If someone presented a solution to us, it would be good—on our own, we cannot find the solution”.—Young person from Community A FGD*

One-off suggestions, that were always underlined with doubt, included upland rice cultivation and using drip irrigation and greenhouses:

*“We could cultivate rice on the plateau or with ramps and pipes connected to water. Once watered, the earth is wet, but the mud does not stay because the sun is beating down. The water disappears but the humidity remains. Apart from that, if we always cultivate in the bas-fonds, I don’t think we can reduce the rate of mosquitoes”.—MRCL*

*“Maybe in a greenhouse...if [rice is grown] in the open air, it's inevitable…Or the rice on the plateau but, for one, I haven’t mastered it and then two, I don’t think our land is fertile enough”.—DKB*

## Discussion

This study aimed to investigate rice farmers’ knowledge, attitudes and perceptions towards mosquitoes and to determine if there are any existing or potential collective riceland vector control initiatives in two rural communities in central Côte d’Ivoire. Most respondents found rice farming complicated because of its prerequisite costs for labour, machinery, inputs, and water, but not necessarily because of the nuisance or diseases caused by mosquitoes. Nonetheless, rice farmers were very familiar with mosquitoes and acknowledged that they caused *djèkouadjo* (malaria) and were less numerous during *harmattan* (dry season). Many farmers believed that mosquitoes originated from grasses or bushes and wastewater within and around villages. Only rice farmers living closer in proximity to the paddies thought that mosquitoes originated from the *bas-fonds*. Despite this, respondents did not identify a link between rice cultivation and mosquitoes; some specified that the rice plant itself did not bring more mosquitoes. Most respondents believed that there were more mosquitoes in recent years than historically because of the construction of the dam, as well as the occurrence of more bushes, garbage, and wastewater. Still, respondents deliberated the importance of the dam and *bas-fonds* for their livelihood and hence, were not convinced that there were solutions to control mosquitoes at farm-level.

Rice farmers in these two Ivoirian communities were knowledgeable about mosquitoes. They were aware that they transmitted malaria, resembling findings reported in rural rice-farming communities in other parts of sub-Saharan Africa, including another area of Côte d’Ivoire, Ghana, Benin, Rwanda, Kenya, and Tanzania [16, 23–25, 27, 34]. Respondents accurately recalled mosquito biting patterns, identifying peak times within a day (from dusk until dawn) and a year. As remarked in other studies, farmers stated that there was a year-round presence of mosquitoes but significantly fewer during the drier periods of a year (i.e., *harmattan* in West Africa) [27, 34]. This corresponds to the fact that *harmattan* is a period where rice is not grown because of the harsh, drought-like conditions, but this study could not directly confirm this because interviews did not explicitly ask about times of rice inactivity. The familiarity of mosquitoes amongst rice farmers alongside the distance of their homes from paddies was correlated with the mosquito control adopted at a household level: farmers from Community A, which was ~ 1 km away from the rice fields, used mosquito nets and insecticidal aerosol sprays whilst farmers in Community B, which was more than 2 km away from rice fields, often used only a bed net and cleaned vegetation around their homes. Farmers in Community B also rarely combined bed net usage with insecticidal sprays or mosquito coils. Otherwise, these methods of vector control have been observed in numerous studies on rice farming communities: it is apparent that since the study by Essé et al*.* in 2002, mainstays of vector control in central Côte d'Ivoire have not changed [23, 26, 34].

Perceptions on historical and current mosquito density were mixed amongst rice farmers in both communities. Whilst the majority attributed recent mosquito increases to nearby dam construction in the last 20–30 years, many participants also attributed them to the presence of more village wastewater and loss of traditions. Traditional/mystic factors (i.e., God or ancestors) were often the believed causes of diseases such as malaria, but this is the first known instance that mosquitoes were also viewed as divine retribution for disobeying or trivializing ancestral practices and prohibitions [23]. These opposing perceptions of increased mosquito incidence could be explained by two reasons. First, before wetlands are converted into irrigation schemes, they also generate many mosquitoes [35, 36]. Thus, rice communities may not have detected a significant difference in mosquito densities before and after wetland conversion/rice cultivation and hence did not attribute it to recent developments in agriculture. Second, recent efforts to universally distribute insecticide-treated nets could have been a confounding factor; many participants rationalized a higher mosquito abundance in the past because of increased protection today from insecticide-treated nets. Conversely, net usage could also have “flattened” mosquito biting peaks, shifting biting to early evening or late morning and causing an increased perception of their presence.

In this study, most rice farmers believed that mosquitoes came from the bushy environment and residual pools of stagnant water in villages. When statements about the link between rice and mosquitoes were sought, most respondents instead acknowledged that the *bas-fonds* (and not the rice fields) contributed to mosquito proliferation. This was surprising because numerous qualitative studies in both East and West Africa have revealed that rice farmers are aware of the impact of irrigated rice cultivation on mosquito production, although some place emphasis on the open canals rather than the rice fields [16, 22, 24, 25, 27, 28, 37]. In the study by Mlozi et al*.* [38] in Tanzania, farmers reported that continuous mosquito breeding was favoured by the following factors: growing rice in bunds (which retained water for long periods of time), poor drainage, and spacing between plants. The findings in our study are similar to those of Benin. Djegbe et al*.* [34] found that despite 94% of rice farmers recognizing stagnant water as breeding sites, only 4% correctly identified rice fields as potential contributors to mosquito production. This means that when spreading awareness or re-educating farmers on this topic (particularly when there are clear mitigation measures to promote), there is a need to distinguish between the different types of stagnant water and the types of mosquitoes associated with them [39]. More attention towards the most productive types of stagnant water for malaria vector breeding is required. Specifically, the differences between *bas-fonds* and rice fields (which is a subset of *bas-fonds*) must be emphasized. Whilst water bodies in wetlands or lowlands are responsible for some *Anopheles* production, it must be highlighted that water from within rice fields are the bigger contributors of malaria vector breeding.

This study illustrates that when farmers were uninformed of, unconvinced of, or indifferent to the link between rice cultivation and malaria, many believed that living with mosquitoes was inevitable. Other farmers viewed the problem as a trade-off between their livelihood and malaria and preferred to suffer the health consequences. Hence, the majority of participants doubted that there was a solution, mainly deeming their existing control methods (environmental management, mostly referring to weeding operations) too ineffective, temporary and/or labour intensive. This was also seen in Kenya, where rice farmers did not apply known vector control methods (e.g., draining stagnant water and clearing vegetation along water canals) due to perceived lack of effectiveness and lack of time to apply [26].

Conversely, once farmers were aware of the rice-mosquito link, they were more motivated to change cultivation practices to minimize mosquito production. This was observed in a handful of respondents from this study who suggested adopting drip irrigation, upland rice cultivation or rice cultivation inside greenhouses. In a qualitative study in Tanzania, farmers seemed not only to take responsibility towards the problem but were highly motivated in solving it [38]. They expressed dissatisfaction towards the government (including agricultural extension workers) for failing to provide them the necessary education to grow rice without intensifying malaria transmission, such as use of *Azolla*(also known as mosquito fern) and intermittent irrigation. In another study in Tanzania, fertiliser-*Bti* mixtures were well-received by rice farming communities who were aware of their impact on mosquitoes. Farmers perceived that the reductions in mosquito densities in their farms (following *Bti* application) enabled extended working hours and that there was a reduced risk of contracting malaria within their household [40]. In this setting, rice farmers were keen to scale up the intervention in terms of area and intervention; they did not think that it was challenging to prepare and apply the mixture (where some reported increased yields) and were willing to contribute to paying for the mixture [40]. It appears that different communities perceive this rice-mosquito issue differently and a variety of approaches can motivate farmers to take up modified rice-growing and mosquito-minimizing methods but in general, improvements on rice yield is the largest determining factor.

Since farmers were unaware of the significance of the rice-malaria link and therefore did not propose many methods of rice growing that could minimize mosquito production, this study was not able to uncover instances of any response to the collective action aspect of riceland mosquito control. However, collective action problems were salient in two observed affairs: bush or weed clearing operations at village-level as mosquito control and at field-level to allow equitable water distribution for rice cultivation. In both instances, communities, in theory, co-operated in “sanitation programmes” to achieve a “common good”. However, in practice, these programmes were often never launched, due to lack of initiative in their leaders, or were unsuccessful. They did not succeed either because of insufficient communication amongst community members (especially amongst rice farmers from different villages who shared an irrigation scheme) or “free-riders”. Individuals then often resorted to.

- Act in their own self-interest but not achieving community goals (e.g., cleaning around their own peri-domestic area),
- Compensate for the “free-riders” (e.g., removing vegetation in the canals for those who did not), or
- Defect further (e.g., farmers visiting the *bas-fonds* at night to divert water to their fields).

Ultimately, farming communities are unable to reach their common objectives. Particularly with regards to water shortage problems in rice cultivation, these collective action problems have led to farmer conflict [41]. Both cases of n-person prisoner’s dilemma are potentially illustrative of issues in organizing farm-level riceland mosquito control: community control may not be achieved if leaders do not show initiative, free-riders exploit the system, and/or if farmers in a rice cooperative are difficult to assemble when they come from different villages.

It is demonstrated that technical solutions (i.e., modified rice-growing methods that minimize mosquito production) must be designed with collective action problems in mind [42, 43].

Perhaps lessons could be learnt from the rice sector’s approach in climate change mitigation. Like malaria vectors, greenhouse gases are harmful emissions that are produced as a side-effect of rice irrigation. In both cases, this happens with little or no awareness on the part of the farmers. Yet, rice-development agencies have been able to scale-up a modified rice cultivation practice, alternate wetting and drying irrigation, amongst farmers in Southeast Asia [44]. Their strategy to facilitate collective action is worth learning from.

The main limitation of our study was the use of double translations (from Baoulé to French and French to English) and involving the data collector KACK only in data interpretation (and not data analysis). The questions that were asked in interviews were not back-translated, which could have helped researchers compare translations for quality and accuracy. This can be problematic as nuance is important in these circumstances. This issue could have been reflected in questions regarding where mosquitoes came from, as it may probe for where mosquitoes fly from, rather than where they may breed. Another limitation of this study was the potential participant bias in asking rice farmers about their views and perspectives on mosquitoes. Although questions were framed open-endedly in order to minimize such bias, farmers could have denied acknowledging links between rice cultivation and mosquitoes (and malaria) based on morality or social acceptability. A third limitation is that despite the larger population in Community B, fewer individuals were recruited because (a) data had reached saturation earlier and (b) fewer individuals practiced rice farming, possibly because it was a relatively more urban community. For this reason, an FGD on youths was not conducted in Community B. Lastly, the data collected on bush and weed clearing, whilst relevant, are still insufficient to make conclusions about the subject of collective action.

## Conclusions

Rice farmers in this part of central Côte d’Ivoire were generally not aware of the link between rice cultivation and malaria vector production. When clear mitigation measures based on agricultural techniques are ready to be adopted, education and training about the rice-mosquito link should not centre around rice farming communities only, but also agricultural and health extension workers. This can come in the form of farmer field schools and training on integrated pest management combined with integrated vector management, as trialled in Sri Lanka [45–47].

First, they should be taught that whilst certain types of stagnant water do generate mosquitoes, specific types (such as rice fields) particularly encourage mosquito proliferation due to the ideal aquatic conditions they present (fresh, clean, sunlit, shallow water with some vegetation). These conditions are most apparent during the first few weeks after transplanting occurs [4]. Second, as pointed out by Djegbe et al*.* [34], farmers and extension workers should be able to recognize mosquito larvae. Third, since farmers did not seem fully informed that mosquitoes could travel from dams or rice fields farther away, they should be taught on migration (i.e., that mosquitoes can fly far distances to find bloodmeal sources). Fourth, regular cleaning of canal vegetation should be emphasized, not only in order to maintain a continuous flow of water (which is unattractive for malaria vector breeding), but also to even out water inequalities amongst fields. Fifth, it is important to emphasize that this association between rice and malaria does not suggest inevitable trade-offs between food security and human health. Instead, this association emphasizes the need for more intersectoral linkages between the agricultural and health sectors in the planning and execution of rice (or any other crop) cultivation. Specifically, it encourages the agricultural sector to take into account of the malaria vectors produced by rice. It encourages the development of modified methods of rice cultivation that can produce good yield, can minimize mosquito proliferation and can eventually be recommended as “good crop husbandry” to farmers [38]. Finally, there needs to be additional effort to avoid free riders in collective actions such as riceland vector control (Additional file 3).

### Supplementary Information

**Additional file 1.** In-depth interview topic guide.

**Additional file 2.** Focus group discussion topic guide.

**Additional file 3.** Images of *bas-fonds* in central Côte d’Ivoire.

## Data Availability

The datasets used and analysed during the current study are available from the corresponding author on reasonable request.

## Abbreviations

FGD: Focus group discussion
IDI: In-depth interview
LSHTM: London School of Hygiene and Tropical Medicine
PAR: Participatory action research
SSA: Sub-Saharan Africa
