# Supplementary material for: Rice farmers’ knowledge, attitudes and practices towards mosquitoes in irrigation schemes in Côte d’Ivoire: a qualitative study
Source: Malar J. 2023 Nov 16;22:352. doi: 10.1186/s12936-023-04785-y (PMC10655379; doi:10.1186/s12936-023-04785-y)
Supplement: Supplementary file 1 — Additional file 1. In-depth interview topic guide. [file 12936_2023_4785_MOESM1_ESM.docx]

# Additional File 1. In-depth interview topic guide

| **Participant ID:** |  | **Participant gender:** | Male / Female |
| --- | --- | --- | --- |
| **Researcher initials:** |  | **Date:** | ____ /____/_____ |
| **Audio file number:** | - start recording - | | |

**Introduction**

I am ­­­­­­­­­­­_______________ from ___________________, collaborating with the London School of Hygiene & Tropical Medicine.

| **Aims of the interview** | *To explore rice communities’ views and perspectives on mosquitoes* |
| --- | --- |
| **Expected duration** | *Approximately one hour* |
| **Why the participant’s cooperation is important** | *We need to know their experiences to find out if, and how, we can reduce mosquito populations in rice farming communities* |
| **What will happen with the collected information?** | *Results will be used to design the next part of the study* |
| **Confidentiality** | *- Inform them -* |
| **Any questions?** | *- Ask -* |
| **Use of tape recorder** | *- Inform them -* |
| **Consent form** | *- Signature -* |

**Demographic and Work History**

Can I ask some details about you and your household?

| **School education level:** |  | **Year of graduation:** |  |
| --- | --- | --- | --- |
| **How long have you lived in this house?** |  | **Are you originally from this area / district?** | Yes / No |
| **How old are you?** |  | | |

**Now, I would like to ask you some questions about your experiences with rice farming and mosquitoes.**

| **Domain** | **Topic and Probes** | |
| --- | --- | --- |
| 1. **Rice farming** | a) | Does your household farm any rice? Who works on the farm?  *Where is the farmland? How long has your family cultivated this land? And of that time, how long has it been rice?* |
|  |  |  |
|  | b) | What kind of rice do you grow?  *Variety, agrosystem (rain-fed/irrigated)* |
|  |  |  |
|  | c) | How easy is it to grow rice?  *What are the difficulties with growing rice? Is water readily available?*  *What are main choices/decisions to be made at the start of the season? What influences those decisions (why choose to do x not y)?* |
|  |  |  |
|  | d) | What are the disadvantages of living next to rice fields?  *Are you busier? What about your health?* |
|  |  |  |
| 1. **Mosquitoes** | a) | Are there mosquitoes here?  *Where? When? What factors influence whether there are many or few?* |
|  |  |  |
|  | b) | Are mosquitoes a problem? What kind of problem? What are the consequences of mosquitoes?  *Are mosquitoes a nuisance?* *How much of a nuisance (compared to flies, bed bugs, …)? Why are mosquitoes a problem? Nuisance? Diseases? Lack of sleep?* |
|  |  |  |
|  | c) | When are mosquitoes a problem? When do they come?   - *What part of the day?* - *Which season(s)?* - *What part of the season?* - *What part of the year?* |
|  |  |  |
|  | d) | The history of mosquitoes: are there more or less now?  *Compared to the last 10 years? Has anything changed? Do you remember a time where it was not a problem? Or a very large problem?* |
|  |  |  |
|  | e) | Where do mosquitoes come from?  *Physically, where?* |
|  |  |  |
| **3. Mosquito control** | a) | Can you control mosquito numbers in the house?  *What can you do in the house to protect yourself? Is there anything you can personally do to control the number of mosquitoes you and your family are exposed to? Ask them for their own ideas.* |
|  |  |  |
|  | b) | If you can control mosquito numbers in the house, do you do them? What do you do? How much does this cost? How effective are they? Is one better than the other?  *If not, why don’t you do them? What would have to happen for you to think about doing these things?* |
|  |  |  |
|  | c) | Can you control mosquito numbers in the community?  *What can we do to reduce the number of mosquitoes in the village? What can an individual rice farmer do?* |
|  |  |  |
|  | d) | If you can control mosquito numbers in the community, do you do them? What do you do?  *If not, why don’t you do them? What would have to happen for you to think about doing these things?* |
|  |  |  |
|  | c) | Can mosquitoes grow in water?  *Is there any change you can make to the way you grow rice that will reduce the number of mosquitoes in your area?* |
|  |  |  |
| **Closing** |  | Is there anything else you think is important in rice farming and mosquitoes that we have not talked about?   - Summarise - Thank participant |
|  |  |  |

**Contact Summary**

| **1** | How would you describe the atmosphere and context of the interview? |
| --- | --- |
|  |  |
| **2** | What were the main points made by the respondent during this interview? |
|  |  |
| **3** | What non-verbal behaviour (gestures and actions) were made throughout the course of the interview? |
|  |  |
| **4** | What new information did you gain through this interview compared to previous interviews? |
|  |  |
| **5** | Was there anything surprising to you personally? Or that made you think differently? |
|  |  |
| **6** | What main messages did you take from this interview about mosquitoes? |
|  |  |
| **7** | Were there any problems with the topic guide (e.g., wording, order of topics, missing topics) you experienced in this interview? |
|  |  |
