# Supplementary material for: Rice farmers’ knowledge, attitudes and practices towards mosquitoes in irrigation schemes in Côte d’Ivoire: a qualitative study
Source: Malar J. 2023 Nov 16;22:352. doi: 10.1186/s12936-023-04785-y (PMC10655379; doi:10.1186/s12936-023-04785-y)
Supplement: Supplementary file 2 — Additional file 2. Focus group discussion topic guide. [file 12936_2023_4785_MOESM2_ESM.docx]

# Additional File 2. Focus group discussion topic guide

| Rice farmers’ views on their responsibility for mosquito production | Where do they think mosquitoes come from? |
| --- | --- |
|  | Does rice generate mosquitoes? |
| Collective initiatives to control mosquitoes, according to the rice farmers | What has been done to control mosquitoes? |
| Prospects for collective solutions to control mosquitoes | What can be done to control mosquitoes? |
| Decision-making process in reducing mosquito production | Why hasn’t anything been done to control mosquitoes? |
