# Supplementary material for: Rice farmers’ knowledge, attitudes and practices towards mosquitoes in irrigation schemes in Côte d’Ivoire: a qualitative study
Source: Malar J. 2023 Nov 16;22:352. doi: 10.1186/s12936-023-04785-y (PMC10655379; doi:10.1186/s12936-023-04785-y)
Supplement: Supplementary file 3 — Additional file 3. Images of bas-fonds in central Côte d’Ivoire. [file 12936_2023_4785_MOESM3_ESM.docx]

# Additional File 3. Images of *bas-fonds* in central Côte d’Ivoire


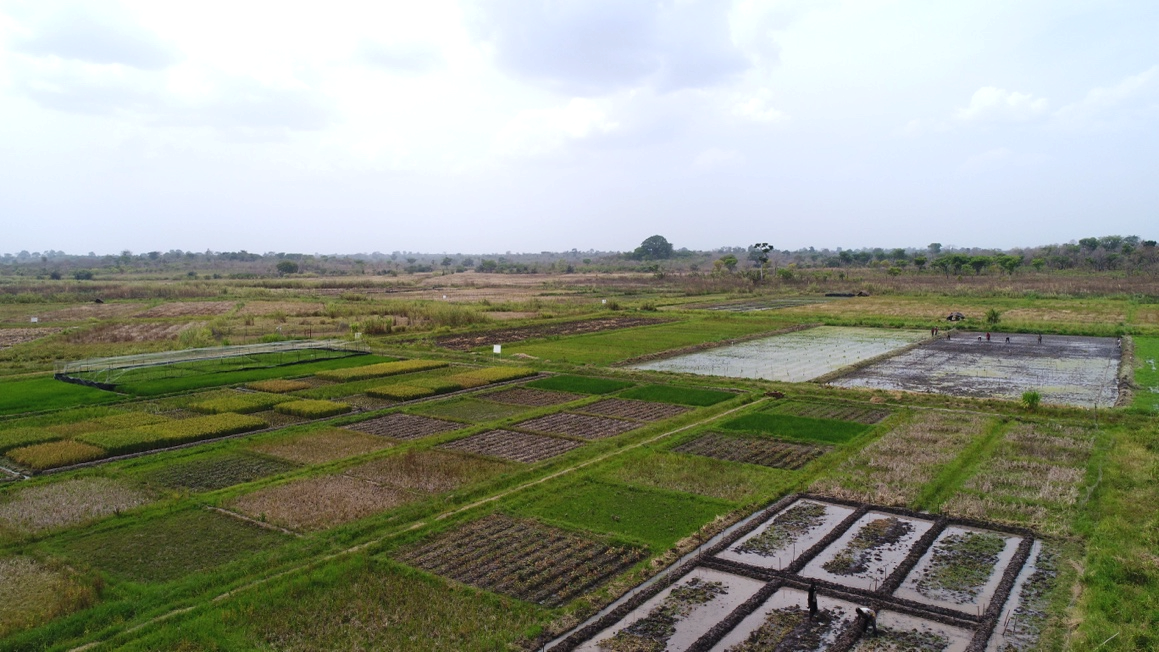


(A) Image of M’bé irrigation scheme taken from a drone


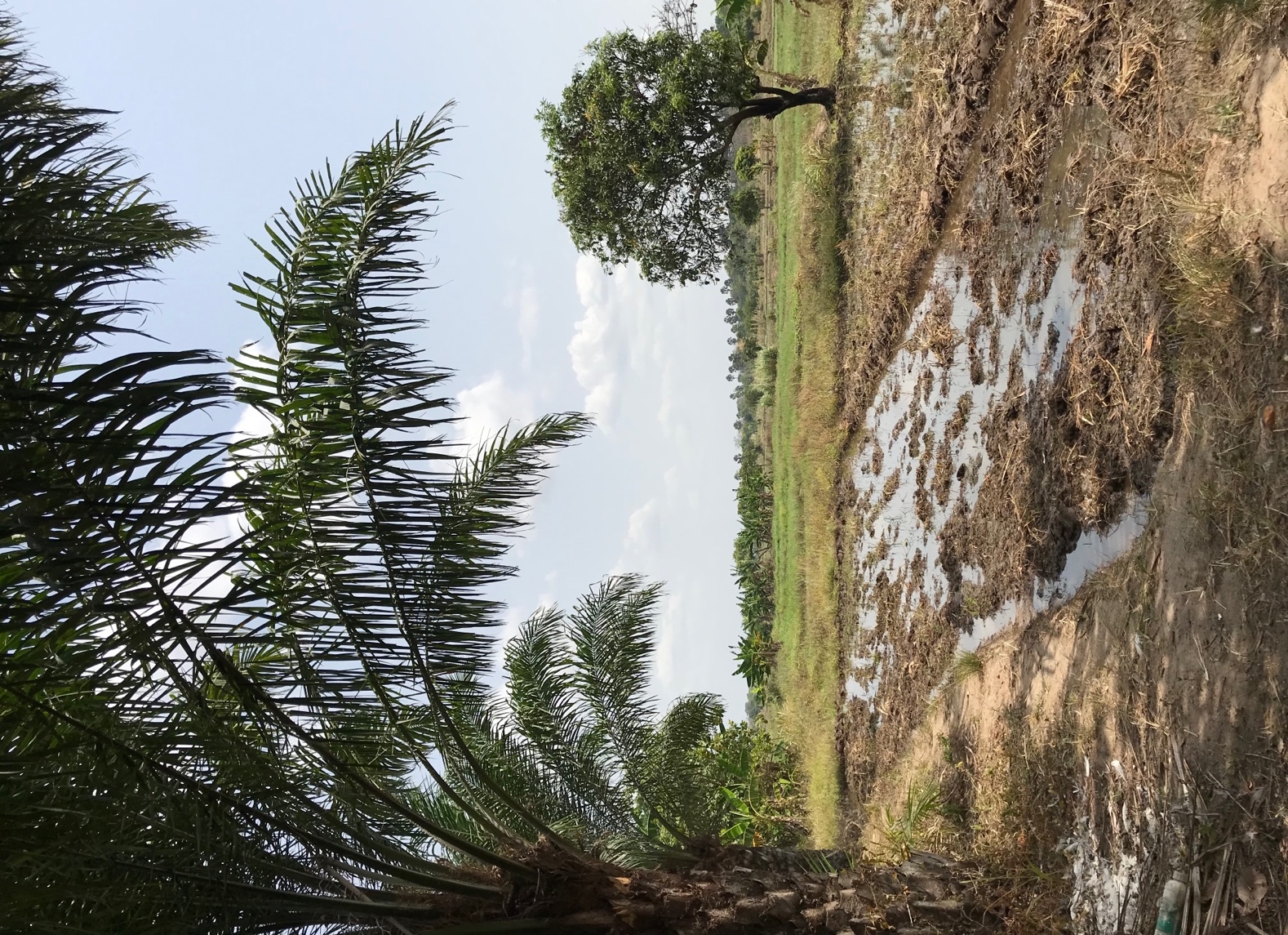


(B) Image of M’bé irrigation scheme taken during the wet season


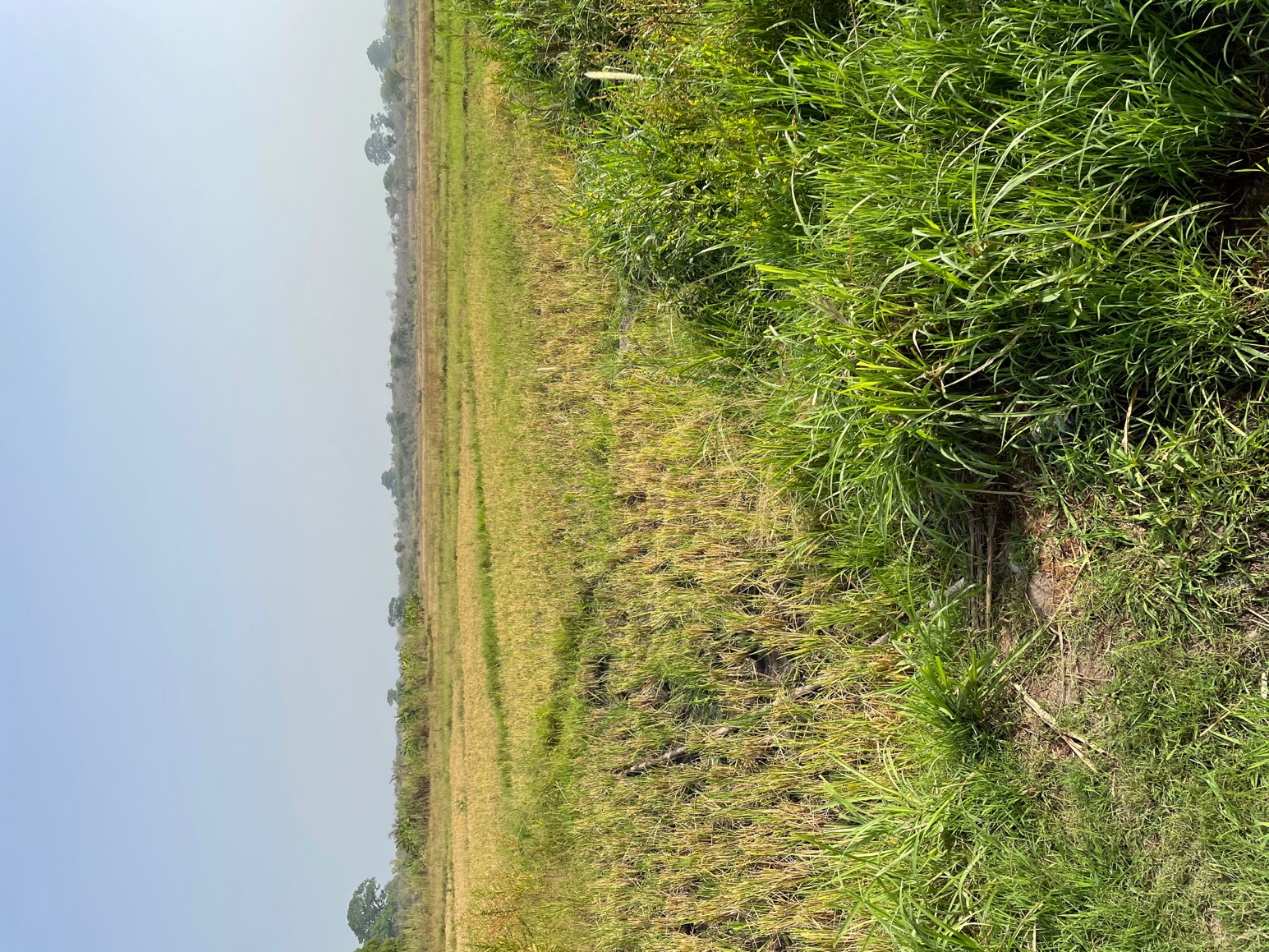


(C) Image of M’bé irrigation scheme taken during the dry season
